# Integrating behavioral dynamics and allee effect in eco-epidemiological model: A comprehensive approach to disease impact

**DOI:** 10.1371/journal.pone.0323928

**Published:** 2025-05-22

**Authors:** K. M. Ariful Kabir

**Affiliations:** Department of Mathematics, Bangladesh University of Engineering and Technology, Dhaka, Bangladesh.; Nova Southeastern University, UNITED STATES OF AMERICA

## Abstract

Predator-prey models in ecology typically focus on direct predation without considering other interactions, such as hunting behaviors on qualitative impact and numerous diseases, which has led researchers to explore the intersection of ecology and mathematics, resulting in the development of mathematical models. This paper introduces a novel eco-epidemiological model that integrates the dynamics of infectious diseases within predator-prey interactions, factoring in direct predation, the roles of infected prey, and the influence of behavioral dynamics. By utilizing evolutionary game theory, this study explores the effects of cooperation and non-cooperation strategies on disease spread and population stability. Numerical simulations reveal that disease transmission rates and the Allee effect significantly impact population stability. Low transmission rates favor stability, while higher rates provoke periodic oscillations that can destabilize the population. The associated controlling cost reduces the risk of infection in prey populations, affecting the predator population. Moreover, the Allee effect exacerbates prey vulnerability, increasing the risk of predator extinction unless disease transmission is curtailed. Findings underscore the importance of considering behavioral dynamics and epidemic factors in conserving species and managing infectious diseases, offering valuable insights into the complex interactions that govern ecosystem health and stability.

## Introduction

Predator-prey relationships are fundamental concepts in ecological research, shedding light on the intricate relationships between species in ecosystems. Most conventional models have primarily concentrated on direct predation, neglecting significant interactions like hunting strategies and the impact of diseases on outcomes. This gap has motivated scholars to explore the intersection of ecology with mathematics, leading to the creation of advanced mathematical models that allow for an in-depth analysis of how populations interact. Studies have highlighted the complex interactions between predators and their prey, emphasizing the importance of developing comprehensive models that capture various ecological factors [1–3]. One critical aspect of these interactions is the impact of infectious diseases on population dynamics, which calls for a deep understanding of how different species manage the threat of diseases to survive [4,5]. Examining the adaptive strategies of affected species to control the spread of these diseases is crucial. These strategies include leveraging dynamics between and within species and environmental signals to exhibit behaviors that avoid infection [6–8]. For instance, some species demonstrate adaptive behaviors like altering their movement patterns to avoid areas with high pathogen loads, engaging in self-isolation or social distancing within their groups, or modifying grooming and foraging habits to minimize contact with contaminated surfaces or infected individuals. Such behaviors are essential for reducing infection risks in populations, thus ensuring species’ continued existence.

Lotka [9] and Volterra [10] laid the cornerstone for extensive research into prey-predator models, crucial for unraveling species interactions within ecosystems [11]. Recent decades have witnessed a surge in investigations into the impact of diseases on these dynamics. Anderson pioneered the exploration of epidemic effects, followed by Anderson et. al. [12], who modified the Lotka-Volterra model to incorporate increased predation rates and halted reproduction in infected prey populations, revealing potential instabilities. Freedman and Hadeler [13] proposed a model where infected prey faced higher predation risks, with predators contracting the disease solely through infected prey consumption. By focusing on transmission through consumption, the model emphasizes the direct role of trophic interactions in disease spread, highlighting the dual impact of infection: increasing prey vulnerability and serving as a vector for predator infection. This approach allows researchers to isolate and analyze the interplay between ecological and epidemiological processes within predator-prey dynamics, providing valuable insights into how infections can cascade through food webs. Haque and Venturino [14] delved into disease transmission within symbiotic communities. Numerous researchers have contributed diverse models focusing on disease transmission within prey populations, enriching the literature [15–20] and others cited.

Investigating the interaction between infected prey and predators is crucial for managing populations, but this component has historically been neglected in theoretical ecology studies. A handful of researchers have recently developed prey-predator models incorporating predator infection [21–23]. Undoubtedly, considering infection in prey populations is more relevant in real-world environments. However, this area has received scant attention, primarily due to the need for mathematical tools to handle the complex differential equations involved in such modeling. Recent efforts, notably in papers [24] and [25], have aimed to address this gap. This study presents a straightforward prey-predator model featuring disease in prey populations. Islam et al. [26] explore an eco-epidemic model focusing on prey infection, including nonlinear refuges, predator harvesting, and Holling type I response. They analyze system stability, reproduction rates, and bifurcations, incorporating environmental noise into a stochastic model. In contrast, using a Holling type-II response, Roy et al. [27] model the dynamics of the Hooghly–Matla estuary, focusing on the interactions among plankton, zooplankton, and fish, with salinity as a crucial environmental factor. Salinity affects the physiological tolerance and distribution of aquatic species and influences the availability of nutrients, altering the productivity of plankton and, consequently, the entire food web. The study underscores how environmental variability directly impacts species interactions and population dynamics by examining stability, Hopf bifurcation, and the salinity-induced changes in fish populations. Both studies support their findings with numerical simulations, providing a deeper understanding of how ecological and environmental factors, such as disease and salinity, shape ecosystem stability. These factors are interconnected because they reveal how intrinsic population dynamics, such as predation and competition, are modulated by extrinsic environmental drivers. This interplay is crucial for predicting ecosystem responses to climate shifts, pollution, or habitat degradation, as it highlights the delicate balance required for maintaining biodiversity and ecosystem functionality.

In response to the limitations of traditional frameworks, this paper introduces a novel model that extends beyond the confines of direct predation to incorporate the dynamics of infected prey. A key innovation lies in including time-varying parameters linked to infection duration, enhancing its applicability to recurring diseases and emerging epidemics. The study further explores the critical influence of the Allee effect, revealing its role in exacerbating prey vulnerability and destabilizing ecosystems under high disease transmission rates. By identifying bifurcations, oscillations, and transitions to chaos, the model offers a deep understanding of how nonlinear interactions and controlling costs shape population dynamics. Its ability to simulate real-world scenarios, such as the impact of immunization strategies or ecological trade-offs, underscores its potential as a powerful tool for conservation biology, ecosystem management, and public health. The findings provide actionable insights into designing targeted interventions, optimizing disease control measures, and maintaining ecological balance, highlighting the model’s broad relevance and transformative impact in addressing complex environmental and epidemiological challenges. A wealth of research has been conducted on how infectious diseases play a role within predator-prey relationships, shedding light on the consequences of such interactions. For example, Pierre et al. [28] put forward a deterministic model that accounts for a disease affecting predator populations. In a similar vein, Xiao and Chen [29] explored how disease dynamics within a prey population can be integrated into predator-prey models. Mukherjee [30] explored a prey-predator model in which the prey population is affected by a microparasite. A Holling type II model characterizes the predator’s functional response. This research investigated predators’ scenarios regarding their dietary preferences and established criteria for persistence when predators target both healthy and infected prey concurrently. Additionally, it addresses the influence of time delays in systems where predators feed on both prey categories. On the other hand, Li and Wang [31] examined a classical stochastic model focused on predators being impacted by disease. By embracing this broader perspective, our model offers a more comprehensive understanding of predator-prey ecosystems, shedding light on the intricate interplay between infectious diseases and population dynamics. The integration of behavioral dynamics, informed by evolutionary game theory, represents a significant advancement in our comprehension of these systems [32–35]. This conceptual framework elucidates the adaptive strategies adopted by species in response to infectious pressures, enriching our understanding of the evolutionary dynamics within predator-prey populations.

The integration of evolutionary game theory has emerged as a robust framework for understanding the intricacies of infectious disease dynamics within epidemiological modeling [36–41]. Rooted in Darwinian principles, this approach aims to elucidate the evolutionary trajectories of various disease transmission and prevention strategies within populations. Within the game-theoretic context, individuals engage in decision-making processes shaped by their interactions with fellow players and their overall fitness considerations. The strategic behaviors of entities can be effectively captured through game-theoretic analysis [42], offering a powerful tool for examining decision-making within complex scenarios. In this framework, individuals, or “players,” are presented with a spectrum of strategies and must select the one that optimizes their desired outcome, or “payoff” [43]. Crucially, these decisions are interdependent, with optimal strategy selection contingent upon the choices made by other participants.

In ecological systems, population growth is often not strictly monotonic with density, and the Allee effect provides a crucial framework for understanding this behavior. The Allee effect describes a scenario where the per capita growth rate or individual fitness increases with population density at low densities. This relationship can manifest in two forms: weak Allee effects, where fitness is depressed but still positive at low population sizes and peaks at intermediate densities before declining due to resource constraints, and strong Allee effects, where fitness becomes negative below a critical density threshold, leading to inevitable extinction if the population size falls beneath this level. Recognizing the role of Allee effects is vital for understanding extinction risks, biological invasions, and population dynamics in small or fragmented populations. It is crucial in predator-prey dynamics, especially when considering disease transmission that can hinder prey recovery at low densities, affecting predators due to reduced food availability and potentially altering disease spread rates. The Allee effect hampers prey recovery at low population densities, as individuals may have difficulty finding mates, resulting in lower reproduction rates. This reduces the available prey for predators and can negatively impact predator populations. Additionally, with fewer prey, diseases spread more easily among the remaining individuals due to increased contact. The effect’s omission in models can lead to overestimating prey resilience and underestimating disease impacts, skewing predictions about ecosystem health. Research highlights include Wang and colleagues’ study [44] on how mating restrictions can trigger the Allee Effect, influencing predator population stability and disease spread, and Kumar et al.’s work [45,46] on the integration of the Allee Effect in food chain models and its significance in predator-prey interactions, revealing its importance in maintaining species coexistence and managing diseases. These studies emphasize the intricate connections between the Allee Effect, disease, and population dynamics, offering insights into ecological management and conservation strategies.

Our research assumes that prey populations possess access to diverse informational resources, encompassing variables such as prey fraction, prevalence of infected individuals, predator presence, and associated costs. Subsequently, they undertake a comparative analysis of specific combinations of these variables to assess the profitability related to different strategies. This evaluative process informs their decision-making regarding potential alterations to their chosen strategies. While the assumption of “perfect information” may present constraints and possibly deviate from real-world practicalities, studying outcomes under such conditions remains a valuable approach for investigating the implications of these assessments on their engagement in disease control measures.

## Model and method

This study proposes a novel computational approach to an eco-epidemiological model incorporating behavioral dynamics. The model elucidates the interactions among three distinct population groups characterized by specific fixed parameters. Within this framework, we introduce an infectious disease that affects the susceptible prey population. Simultaneously, predators in the ecosystem consume both healthy and diseased prey. Central to this model are the symbols S(t) and I(t), representing the susceptible and infected prey populations, respectively, while P(t) denotes the predator population. The relationships among these variables are mathematically defined through a set of nonlinear differential equations (Table 1), as detailed in reference [47].

**Table 1:** Variable and parameter with details descriptions.

Variables/ Parameters	Description
S	Susceptible prey
I	Infected Prey
P	Predator
r	Growth rate of the susceptible prey
b	Intra-class competition of susceptible prey
c	Inter-class effect infected on susceptible
β(x)	Dynamic rate of disease transmission
$\alpha_{1}$	Attack rate of predator on susceptible prey
$\alpha_{2}$	Attack rate of predator on infected prey
$c_{1}$	Transfer efficacy of predator on susceptible prey
$c_{2}$	Transfer efficacy of predator on infected prey
d	Half-saturation constant of predator for infected prey
σ	Half-saturation constant of predator for susceptible prey
a	Half-saturation constant for disease transmission
μ	Death rate of infected prey
θ	Allee parameter
m	Natural death rate of predator
$\beta_{0}$	Baseline transmission rate
x	Dynamics of the strategy
e	Proportionality constant of the cooperator
$C_{S}$	Associated controlling cost of susceptible Prey
$C_{P}$	Associated controlling cost of Predator


$$\frac{dS}{dt}=\left[r-bS(t)-cI(t)-\frac{\beta (x)I(t)}{a+S(t)}-\frac{\alpha_{1}P(t)}{\sigma +S(t)}\right]S(t),$$ (1.1)

$$\frac{dI}{dt}=\left[\frac{\beta (x)S(t)}{a+S(t)}-\frac{\alpha_{2}P(t)}{d+I(t)}-\mu \right]I(t),$$ (1.2)

$$\frac{dP}{dt}=\left[\left(\frac{c_{1}\alpha_{1}S(t)}{\sigma +S(t)}+\frac{c_{2}\alpha_{2}I(t)}{d+I(t)}\right)\frac{P(t)}{\theta +P(t)}-m\right]P(t).$$ (1.3)

The model is subject to initial conditions specified as $(S(0),\;I(0),\;P(0))=(S_{0},I_{0},\;P_{0})\geq 0$. Here, r denotes the growth rate of the susceptible prey, b represents the intra-class competition among the susceptible prey, and c signifies the inter-class competition between the susceptible and infected prey populations. Here, a saturated incidence rate $\frac{\beta I}{a+S(t)}$ is considered, where βI represents the infection force of the disease, and $\frac{1}{a+S(t)}$ captures the inhibitory effect due to behavioral changes in susceptible individuals as their population increases. This formulation reflects how a growing number of susceptibles leads to a reduction in the transmission rate. Additionally, a Holling type-II functional response is utilized by predators to capture prey, accurately representing the predator-prey interactions in the model. They capture susceptible prey at a rate of $\frac{\alpha_{1}S(t)}{\sigma +S(t)}$ and infected prey at a rate of $\frac{\alpha_{2}I(t)}{d+I(t)}$. The parameters $c_{1}$ and $c_{2}$ describe the predator’s efficiency in converting captured susceptible and infected prey into nutrition. The mortality rates for infected prey and predators are denoted by μ and m, respectively. The term $\frac{P(t)}{\theta +P(t)}$ represents the weak Allee effect function [48,49], where θ is the Allee parameter. A higher value θ corresponds to a more significant Allee effect, reducing the predator’s per capita growth rate. The weak Allee effect is incorporated into population growth models by adjusting the birth term of the non-Allee growth equation with a probability function, denoted as $\frac{P(t)}{\theta +P(t)}$. This function represents the probability that a female successfully finds and mates with at least one male during the reproductive period. Further biological justifications for this approach can be found in [50] and [51]. The probability function $\frac{P(t)}{\theta +P(t)}$ must meet the following fundamental conditions: (1) No mating occurs when the population size is zero, (2) As population size increases, the probability of successful mating also increases, (3) At large population sizes, mating is virtually guaranteed.

The term β(x)I(t) describes the infection force of the disease, where β(x) reflects the dynamic transmission rate influenced by behavioral dynamics [52]. A game-based approach to simulating the transmission rate of diseases, symbolized by β(x), is proposed. It is presupposed that the cooperation level of an individual, indicated by the variable x [0,1], reflects their compliance with control measures. Generally, species that follow these behaviors have a lower disease transmission rate. Thus, the effects of adopting specific strategies in this game on epidemiological trends are aimed to be investigated. A model will be developed where the infection rate is contingent upon the strategy selected in the game, as will be described in the following sections to link game theory with epidemiological analysis.

### Behavioral dynamics

The integration of evolutionary game theory into eco-epidemiological modeling offers a meticulous understanding of infectious disease dynamics. This approach explores the evolution of disease transmission and prevention strategies through a game-theoretic lens. By analyzing the decisions aspect, the framework highlights how populations assess various factors—like disease prevalence and predator presence—to adjust their strategies for optimal outcomes [35].


$$\beta (x)=\beta_{0}(1-ex).\;$$ (2)

$$\dot{x}=x(1-x)\left[\;-C_{S}S+I(t)+C_{P}P\right].\;$$ (3)

The integration of evolutionary game theory into eco-epidemiological modeling offers a comprehensive framework for understanding how populations adjust their strategies in response to varying conditions, such as disease prevalence and predator presence. The given mathematical equations provide insight into this adaptive process. Equation (2) represents the transmission rate of the disease as a function of the strategy x, where $\beta_{0}$ is the baseline transmission rate, 1−ex indicates the reduction in transmission due to preventive measures, and e(0≤e≤1) implies the proportionality constant of the cooperator. As more effort or resources are allocated towards preventing disease (higher x), the effective transmission rate β(x) decreases.

Equation (3) describes the dynamics of the strategy x, illustrating how it evolves over time. The term $\dot{x}$ represents the time derivative of x, capturing the rate of change in the strategy. The factor x(1−x) ensures that x remains within the interval [0,1], reflecting frequency-dependent selection dynamics. The components $-\;C_{S}S(t)$, I(t), and $C_{P}P(t)$ represent the influences of the susceptible prey population, the infected prey population, and the predator population, respectively. The coefficients $C_{S}S$ and $C_{P}P$ scale the impact of the susceptible prey and predator populations on the strategy, respectively. Here, $C_{S}$ and $C_{P}$ are fixed constants that range from 0 to 1. These parameters are not based on empirical data or field experiments but are instead chosen as abstract measures of the relative cost or pressure exerted by disease transmission $\left(C_{S}\right)$ and predation $\left(C_{P}\right)$. The fixed values allow for a controlled exploration of how these factors influence the strategy dynamics in the model, providing a theoretical framework to understand the interplay between disease and predation in shaping population behaviors. Populations adjust their strategies dynamically by balancing the costs associated with disease prevention and the risks of predation, aiming to find an optimal strategy x that minimizes disease transmission while considering predation impacts. The fixed ranges for $C_{S}$ and $C_{P}$ ensure the model remains generalizable while highlighting how variations in these parameters drive the evolution of x. This framework captures the adaptive nature of populations in navigating complex ecological and epidemiological challenges.

**Fig 1:**
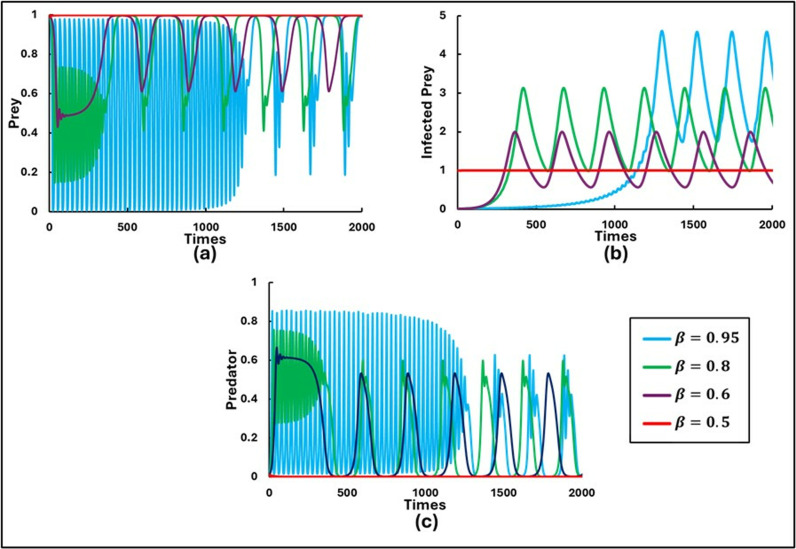
Illustrations of the sequential trends in the populations of **(a)** Prey, **(b)** Infected Prey, and **(c)** Predator are presented for varying levels of the disease transmission rate ($\beta_{0}\;=\;0.5,\;0.6,\;0.8,\;0.95$). The other parameters are set as follows: $a=0.3636,\;b=1.0,\;\alpha_{1}=0.01,\;\sigma =15,\;c=0.01,\;r=1.0,\;\alpha_{2}=0.05,\;d=0.5,\;\mu =0.4,\;c_{1}=2,\;\theta =0,\;c_{2}=1.0,\;e=1.0,\;x=0.0$ and  m=0.01 [47].

**Fig 2:**
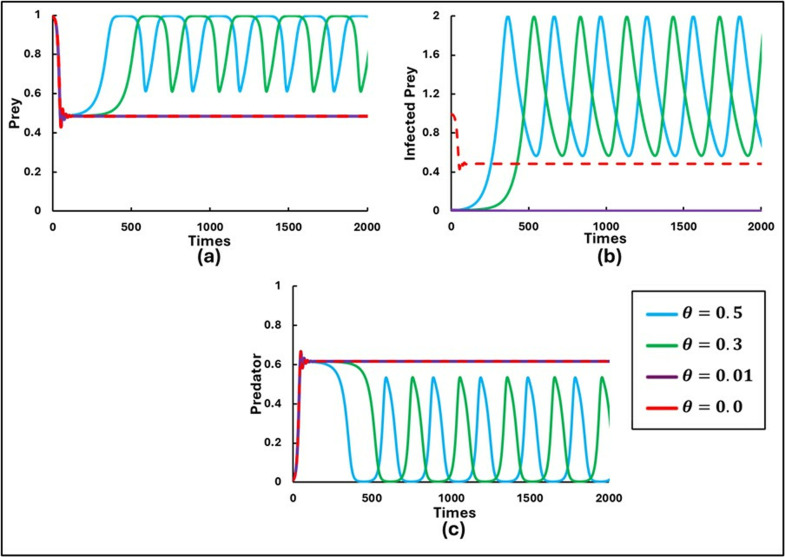
Illustrations of the sequential trends in the populations of **(a)** Prey, **(b)** Infected Prey, and **(c)** Predator for $\beta_{0}\;=\;0.7$ are presented for varying levels of the Allee parameters (θ = 0.0, 0.01, 0.3, 0.5). The other parameters are set as follows: $a=0.3636,\;b=1.0,\;\alpha_{1}=0.01,\;\sigma =15,\;c=0.01,\;r=1.0,\;\alpha_{2}=0.05,\;d=0.5,\;\mu =0.4,\;c_{1}=2,\;c_{2}=1.0,\;e=1.0,\;x=0.0$ and  m=0.01 [47].

**Fig 3:**
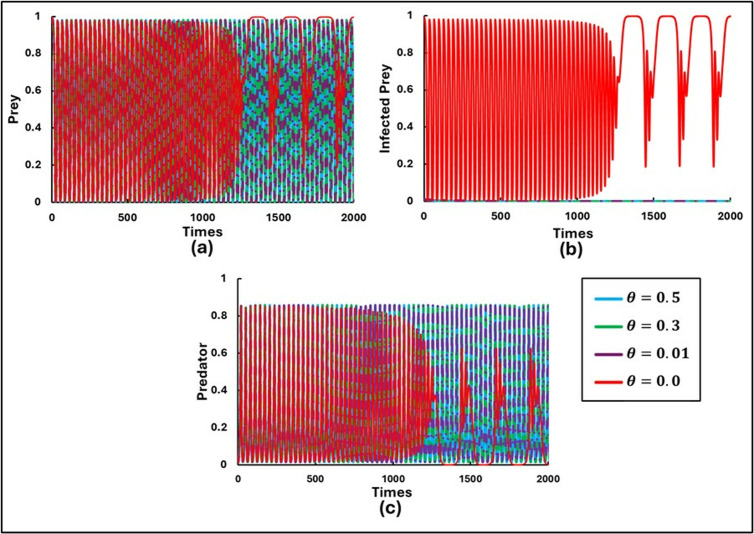
Illustrations of the sequential trends in the populations of **(a)** Susceptible Prey, **(b)** Infected Prey, and **(c)** Predator for $\beta_{0}\;=\;0.95$ are presented for varying levels of the Allee parameters (θ = 0.0, 0.01, 0.3, 0.5). The other parameters are set as follows: $a=0.3636,\;b=1.0,\;\alpha_{1}=0.01,\;\sigma =15,\;c=0.01,\;r=1.0,\;\alpha_{2}=0.05,\;d=0.5,\;\mu =0.4,\;c_{1}=2,\;c_{2}=1.0,\;e=1.0,\;x=0.0$ and  m=0.01 [47].

To explore the findings and study the rich dynamics of the system (1.1–3), extensive numerical simulations were performed. These simulations were conducted using C++ and COLAB (Python), with suitably chosen small time steps for the finite difference method. The set of parameters used for the numerical simulations were as follows: $a=0.3636,\;b=1.0,\;\alpha_{1}=0.01,\;e=15,\;c=0.01,\;r=1.0,\;\alpha_{2}=0.05,\;d=0.5,\;\mu =0.4,\;c_{1}=2,\;\theta =0,\;c_{2}=1.0,$ and  m=0.01. Initially, the scenario without control strategies and the Allee effect (x=0 and θ=0) was considered by varying β=0.5, 0.6, 0.8, and 0.9with initial values [S(0),I(0),P(0rbrack=[0.99,0.01,0.01], as shown in Fig 1. Further, Figs 2 and 3 illustrate the model’s outcomes for $\beta_{0}$ values of 0.7 and 0.95, respectively, while considering various Allee effects (θ= 0.0, 0.01, 0.3, and 0.5). Both figures are subdivided into three panels, depicting distinct outcomes: (a) Susceptible Prey, (b) Infected Prey, and (c) Predator.

## Result and discussion

An innovative eco-epidemiological model has been introduced in this study, integrating behavioral dynamics into a prey-predator framework where the susceptible prey can be infected. The combination of epidemic and prey-predator interactions is pioneered, with disease occurrences, behavioral patterns, periodic characteristics, and stability dynamics illustrated through numerical simulations. Time-varying parameters based on infection duration distributions are featured in the model, and dilemma scenarios are extended to include periodic behaviors. This approach enhances the forecasting of disease spread in populations with ongoing immunization, aiding in planning optimal disease control strategies. The versatility of the proposed model is demonstrated, applying to both recurring diseases with existing immunization measures and unforeseen diseases. Non-linear behaviors exhibited by species during an epidemic are accounted for in the model. Game theory, specifically evolutionary game theory (EGT), is employed to model these behaviors, focusing on cooperation and non-cooperation within the population during an epidemic. By integrating strategies for reducing or controlling behaviors into the EGT model within the eco-epidemiological framework, the impact of these behaviors on disease transmission can be described and analyzed. The relationship between changes in infection rates and prevailing behaviors is elucidated, emphasizing the role of eco-epidemiological information in influencing behavioral choices.

Fig 1 illustrates the numerical solutions of the model over time without considering behavioral dynamics and the Allee effect. The figure is divided into three parts, depicting different outcomes: (a) Susceptible Prey, (b) Infected Prey, and (c) Predator. The dynamical behaviors of the model were observed by varying the infection rate, β, in the absence of control strategies and the Allee effect. When $\beta (x)=\beta_{0}=0.5\;(x=0)$, the system exhibits a stable focus around the coexisting equilibrium, indicating stability. As the rate of disease transmission gradually increases beyond this threshold value, the system transitions to an unstable state and displays periodic solutions. Specifically, for $\beta_{0}=0.5$, the system remains stable, in which susceptible prey and infected prey exist. However, with further increases in the rate of disease transmission, the system exhibits higher periodic oscillations for$\;\beta_{0}=0.6,\;0.8$, and 0.9. This demonstrates that the system’s stability is compromised as the infection rate increases, leading to more pronounced oscillatory behavior.

In Fig 2, it is observed that the populations of Susceptible Prey, Infected Prey, and Predator exhibit a stable focus around the equilibrium for scenarios without the Allee effect (θ = 0) and with a lower value of θ (= 0.01), indicating system stability. This stability suggests that, in the absence of a higher Allee effect, the susceptible prey population can maintain a sufficient size to support healthy and infected individuals and their predators without drastic fluctuations. However, as the value of θ increases, indicating a more substantial Allee effect, the system demonstrates more pronounced periodic oscillations. This increase in oscillations indicates that higher Allee effects make the susceptible prey population more vulnerable to destabilization, causing fluctuations in the populations of infected prey and predators.

Fig 3 presents a scenario with a higher disease transmission rate ($\beta_{0}=\;0.95$). Here, infected prey persist only without the Allee effect (θ=0). Infected prey is absent for both minimal and higher values of θ. This absence suggests that when the disease transmission rate is high, even a slight Allee effect can lead to the eradication of the infected prey population. Nevertheless, the populations of susceptible prey and predator continue to exist, displaying an oscillating tendency. This oscillation indicates that despite the absence of infected susceptible prey, the Allee effect still influences the interplay between the susceptible prey and predator populations, leading to fluctuations.

Introducing the Allee effect (θ) into the system reveals that a higher value of θ corresponds to a more pronounced Allee effect, thereby increasing the critical population size required for growth. When θ is set to values such as 0.0, 0.01, and 0.3, the extinction of the infected prey population is observed. As the θ is further increased, it is noted that the system exhibits periodic solutions, with θ = 5.0 serving as a key threshold. As the parameter θ gradually increases, the system undergoes a series of transition instability. Initially, chaotic oscillations are present, which then shift to period-doubling behavior. This period-doubling eventually gives way to limit cycle oscillations, and finally, the system stabilizes into a stable focus. In contrast, the dynamics of the susceptible prey and predator populations exhibit chaotic oscillations throughout, with increasing values of θ leading to a faster rate of oscillation.

For instance, consider a population of a particular fish species inhabiting a lake, which serves as prey for a larger predatory fish species. The Allee effect significantly influences the prey population’s dynamics, which characterizes the challenges populations face when their numbers fall below a critical threshold. If the susceptible prey fish experiences a strong Allee effect (high θ), its population faces a precarious situation. Overfishing or environmental disturbances could reduce its numbers to a point where recovery becomes nearly impossible. Below this critical threshold, several synergistic challenges emerge: the reduced density hinders effective mate finding, leading to diminished reproduction rates; smaller groups are less able to maintain collective behaviors such as schooling, which are critical for predator avoidance; and weakened genetic diversity increases vulnerability to diseases or environmental changes. These factors compound, pushing the population toward local extinction. Conversely, when the Allee effect weakens (low θ), the prey fish population demonstrates resilience. Smaller groups can still achieve positive growth rates even if their numbers decline due to predation or other pressures. Behavioral adaptations, such as flexible foraging or enhanced vigilance, and ecological advantages, like lower competition for resources in smaller populations, allow them to recover over time.

The results illustrated in Figs 2 and 3 highlight the profound implications of the Allee effect on the stability and persistence of ecological communities. A strong Allee effect amplifies the risk of population collapse for susceptible prey while indirectly affecting the infected prey and predator populations. For instance, a decline in susceptible prey numbers could force predators to shift their diet, intensifying pressure on other species or disrupting the ecological balance. Conversely, in systems with a weaker Allee effect, the interconnected dynamics between susceptible prey, infected prey, and predators are more likely to stabilize, fostering long-term coexistence. This deeper analysis underscores the importance of considering the Allee effect in ecological modeling, as it not only shapes the direct outcomes for individual species but also cascades through the entire ecosystem, influencing trophic interactions and community dynamics. By understanding these nuances, conservation strategies can be better tailored to address critical thresholds and enhance population resilience under environmental or anthropogenic pressures. A more substantial Allee effect leads to greater vulnerability and potential destabilization, whereas a small Allee effect allows for more resilient population dynamics.

**Fig 4:**
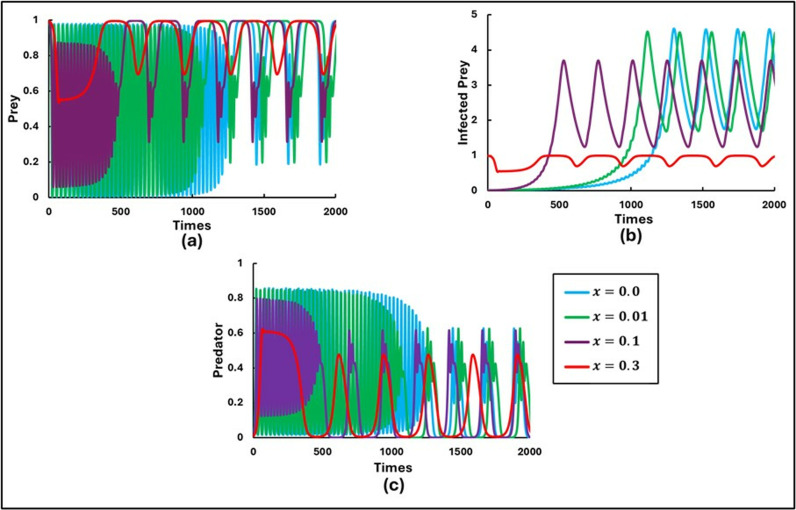
Illustrations of the sequential trends in the populations of **(a)** Prey, **(b)** Infected Prey, and **(c)** Predator for $\beta_{0}\;=\;0.95$ are presented for varying levels of the controlling behavioral parameters (x = 0.0, 0.01, 0.1, 0.3). Here, x is presumed as fixed values (like constant) not the dynamic. The other parameters are set as follows: $a=0.3636,\;b=1.0,\;\alpha_{1}=0.01,\;\sigma =15,\;c=0.01,\;r=1.0,\;\alpha_{2}=0.05,\;d=0.5,\;\mu =0.4,\;c_{1}=2,\;c_{2}=1.0,\;e=1.0,\;\theta =0.0$ and  m=0.01 [47].

Fig 4 illustrates the outcomes for different values of the transmission rate reduction parameter x (0.0, 0.01, 0.1, and 0.3), with a fixed $\beta_{0}$, θ=0.0 and e=1.0. The system exhibits periodic unstable oscillations across all values of x. However, a detailed examination reveals that increasing x to 0.3 significantly decreases the number of infected preys. This reduction occurs because higher values of x represent a more robust response from the susceptible prey species to control the spread of disease. In ecological terms, when x is increased, it implies that susceptible prey species are actively gathering more information from their environment and adopting behaviors that help them avoid infection. This could involve avoiding areas with high infection rates, improving their immune responses, or other mechanisms to reduce transmission. Consequently, the disease spread is mitigated, leading to fewer infected individuals within the susceptible prey population.

Moreover, the effect of higher x values is also evident in the oscillatory behavior of the system. With higher x, the oscillations of the number of infected preys become slower. This means that the overall number of infected prey decreases, and the system stabilizes to some extent, reducing the frequency and amplitude of the oscillations. This stabilization occurs because the proactive measures taken by susceptible prey lead to a more controlled and less volatile dynamic infection. Increasing the transmission rate reduction parameter x leads to a significant decrease in the number of infected prey and results in slower, more stable oscillations in the system. These findings highlight the significance of proactive disease management behaviors in reducing infection rates and stabilizing population dynamics.

**Fig 5:**
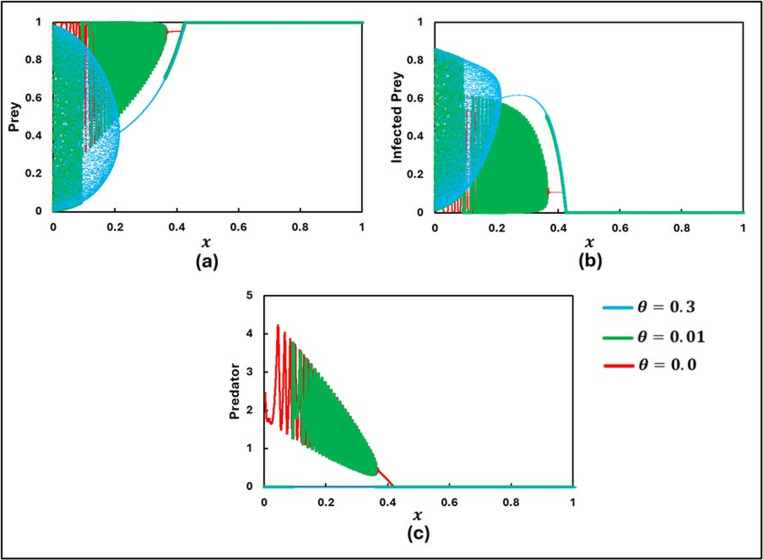
The figure presents a bifurcation diagram depicting equilibria, oscillatory dynamics, and chaotic behavior about the parameter x. The subpanels **(a)**, **(b)**, and (c) show the trends for Susceptible Prey, Infected Prey, and Predator, respectively. Three different values for the Allee parameter θ are considered: θ=0.0, θ=0.01, and θ=0.03. The other parameters are set as follows: $a=0.3636,\;b=1.0,\;\alpha_{1}=0.01,\;\sigma =15,\;c=0.01,\;r=1.0,\;\alpha_{2}=0.05,\;d=0.5,\;\mu =0.4,\;c_{1}=2,\;\theta =0,\;c_{2}=1.0,\;e=1.0,$ and  m=0.01 [47].

Fig 5 presents the one-parameter bifurcation diagram along x, considering the absence of the Allee effect (θ=0.0) and without incorporating behavioral aspects. The analysis reveals that with a gradual increase in x, the system transitions to a stable equilibrium. Notably, bifurcating periodic solutions originating from the coexisting equilibrium point are stable for x values exceeding 0.42. No unstable or oscillatory conditions are observed in these scenarios, regardless of the θ values.

Interestingly, for higher θ values, the infected prey population remains nearly constant across all cases, eventually converging to zero. This finding underscores that increased x values, representing higher levels of disease control strategies, lead to a more significant proportion of protection from infections. Conversely, lower x values are associated with increased oscillatory conditions. When the Allee parameter θ is increased to 0.3, there is a noticeable increase in prey infection compared to lower θ values. The heightened Allee effect at higher θ values can precipitate the extinction of predator species. As the Allee effect causes the susceptible prey population to decline, predators struggle to find sufficient food. This food scarcity leads to a decline in the predator population. Although this reduction in predator numbers might temporarily alleviate pressure on the susceptible prey, the susceptible prey population cannot recover if it remains below the critical threshold due to the Allee effect. Consequently, the predator population faces extinction as their primary food source becomes inadequate.

In a prey-predator model where the prey population is susceptible to infection, the disease transmission rate is critical in shaping the system’s dynamics. Decreasing the disease transmission rate results in fewer infected prey over time, significantly enhancing the overall health and size of the susceptible prey population. Predators can better meet their dietary needs with more healthy susceptible prey available, potentially supporting a larger or more stable predator population. Furthermore, if predators prey on healthy individuals preferentially, a lower infection rate means that the remaining infected prey might experience less predation pressure, indirectly benefiting their survival. Consequently, decreasing the disease transmission rate leads to a healthier and more robust susceptible prey population, with fewer individuals infected. This can result in a larger and more stable prey population, providing predators with a more reliable food source. As a result, predator populations may also become more stable or increase in size. Overall, the system’s dynamics become less volatile, and the reduced disease prevalence contributes to a more balanced and resilient ecosystem.

**Fig 6:**
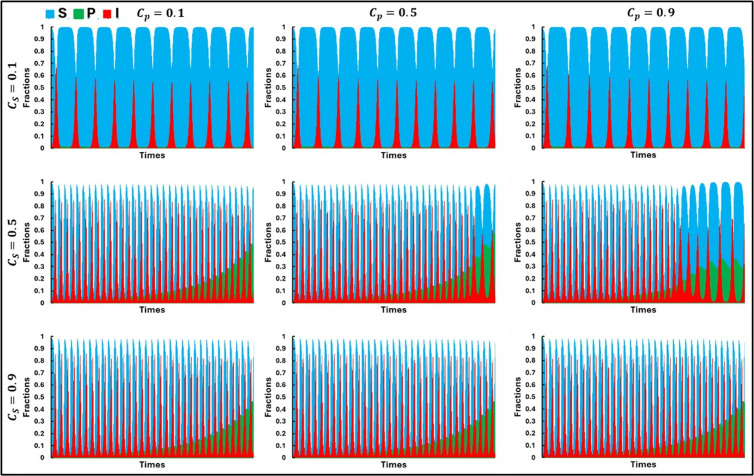
Illustrations of the sequential trends in the populations of **(a)** Prey, **(b)** Infected Prey, and **(c)** Predator by considering behavioral dynamics for varying levels of the associated costs ($C_{S}\;=\;0.1,\;0.5,\;0.9$) and ($C_{P}\;=\;0.1,\;0.5,\;0.9$). The other parameters are set as follows: $a=0.3636,\;b=1.0,\;\alpha_{1}=0.01,\;\sigma =15,\;c=0.01,\;r=1.0,\;\alpha_{2}=0.05,\;d=0.5,\;\mu =0.4,\;c_{1}=2,\;c_{2}=1.0,\;e=1.0,\;\theta =0.0$ and  m=0.01 [47].

Fig 6 presents the numerical solutions of the prey-predator infectious model with behavioral dynamics over time, examining various combinations of controlling costs for susceptible prey $\left(C_{S}\right)$ and predators $\left(C_{P}\right)$. As the associated controlling cost for susceptible prey $\left(C_{S}\right)$ increases from lower values $\left(C_{S}=0.1\right)$ to higher values $\left(C_{S}=0.9\right)$, a noticeable progression in the number of infected preys is observed, accompanied by the emergence of cyclic behavior evolving into more chaotic dynamics. This is because lower $C_{S}$ values indicate minimal investment in infection control, leading to moderate infection rates and stable periodic oscillations. In contrast, higher $C_{S}$ values represent a significant investment, potentially causing unintended consequences such as increased pathogen resistance or higher fitness costs, resulting in chaotic dynamics due to severe stress on the susceptible prey population. On the other hand, increasing the controlling cost for predators $C_{P}$ leads to a reduced tendency for change, with higher $C_{P}$ values minimizing or relaxing periodic oscillations. This trend is discernible in intermediate cases, such as when $C_{S}=0.5$, where moderate control measures induce more noticeable periodic behaviors. The higher $C_{P}$ values imply that predators invest more in managing their interactions with susceptible prey, stabilizing their food sources, and reducing predation pressure, which leads to more stable system dynamics. These findings illustrate the delicate balance between infection control costs and population dynamics in a prey-predator system, highlighting that while increasing $C_{S}$ can lead to more complex and chaotic dynamics, increasing $C_{P}$ tends to stabilize population oscillations, thereby maintaining ecosystem stability.

**Fig 7:**
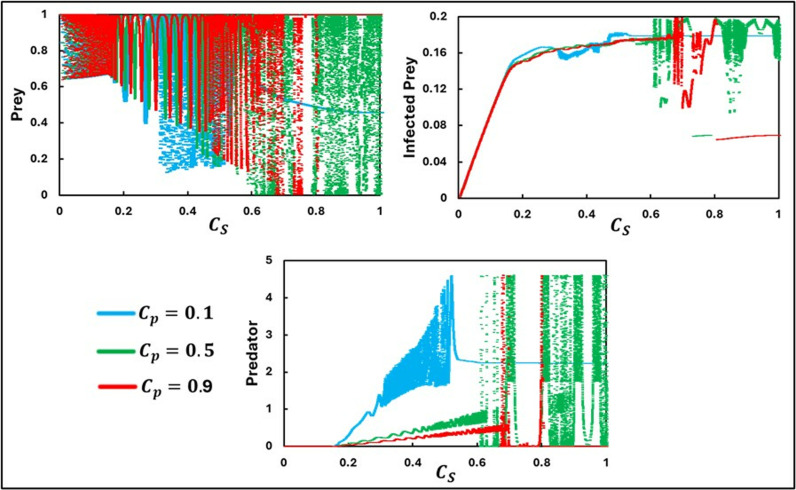
The figure presents a bifurcation diagram depicting equilibria, oscillatory dynamics, and chaotic behavior about the parameter $C_{S}$. The subpanels **(a)**, **(b)**, and (c) show the trends for Prey, Infected Prey, and Predator, respectively. Three different values for the cost $C_{P}$ are considered: $C_{P}=0.1,\;C_{P}=0.5$, and $C_{P}=0.9$. The other parameters are set as follows: $a=0.3636,\;b=1.0,\;\alpha_{1}=0.01,\;\sigma =15,\;c=0.01,\;r=1.0,\;\alpha_{2}=0.05,\;d=0.5,\;\mu =0.4,\;c_{1}=2,\;\theta =0,\;c_{2}=1.0,\;e=1.0,\;\theta =0.0,\;$and  m=0.01 [47].

Fig 7 presents a specific temporal scenario illustrating the dynamics of susceptible prey, infected prey, and predators, as influenced by varying the associated controlling cost for susceptible prey $\left(C_{S}\right)$ and different values of the controlling cost for predators $\left(C_{P}=0.1,0.5,0.9\right)$. This figure highlights the complex interplay between predator-prey interactions, incorporating defensive attributes against disease and behavioral changes in prey species. As $C_{S}$ increases along the x-axis, prey disease incidence shows a notable rise. The equilibrium state of the infected prey transitions from stable to unstable conditions, with higher $C_{S}$ values leading to increased oscillatory behavior. Additionally, the impact of $C_{P}$ on the system dynamics is significant. At higher $C_{S}$ values, susceptible prey, and predator populations exhibit periodic behavior when $C_{P}$ is set at 0.5 and 0.9. Conversely, lower $C_{P}$ values (i.e., $C_{P}=0.1$) reveal three distinct responses to increasing $C_{S}$. Specifically, intermediate $C_{S}$ values induce chaotic dynamics, whereas lower and higher $C_{S}$ values result in stable population dynamics.

The transitions depicted in Fig 7—from stable equilibria to periodic oscillations and chaotic behavior—illustrate the profound sensitivity of predator-prey systems to the controlling costs associated with both susceptible prey $\left(C_{S}\right)$ and predators $\left(C_{P}\right)$. As $C_{S}$ increases, the defensive investments by susceptible prey amplify, likely diverting resources from reproduction or other survival functions. This trade-off leads to a rise in disease incidence, destabilizing the equilibrium and triggering oscillatory behavior in population sizes. The periodicity observed at intermediate $C_{S}$ values reflect the system’s attempt to balance disease control and predation pressure. At higher values of $C_{P}$ (e.g., $C_{P}=\;0.5,\;0.9$), the predators face increased energetic or behavioral costs in managing their interactions with prey, potentially altering their hunting efficiency or reproductive success. These changes, in turn, modulate the oscillatory and periodic patterns seen in prey populations. In contrast, lower $C_{P}$ values $\left(C_{P}\;=\;0.1\right)$ allow for chaotic dynamics at intermediate $C_{S}$, highlighting a critical threshold where minor adjustments in prey behavior lead to disproportionately large and unpredictable changes in the ecosystem.

These observations underscore the intricate nature of predator-prey systems, where varying the controlling costs for susceptible prey and predators can lead to a wide range of dynamic behaviors, from stability to chaos, emphasizing the importance of balanced management strategies in ecological models. The model shows that as the controlling cost for susceptible prey increases, there is a notable rise in the incidence of disease among prey, leading to transitions from stable to unstable equilibrium states and increased oscillatory behavior. This suggests that higher investments in disease control by prey can inadvertently destabilize the population dynamics. In natural ecosystems, such dynamics can be observed in how species invest in defense mechanisms and how predators adapt their strategies, influencing the overall stability and health of the ecosystem. Therefore, effective management and conservation strategies must consider these complex interactions and the potential for stabilizing and destabilizing effects to maintain ecological balance.

**Fig 8:**
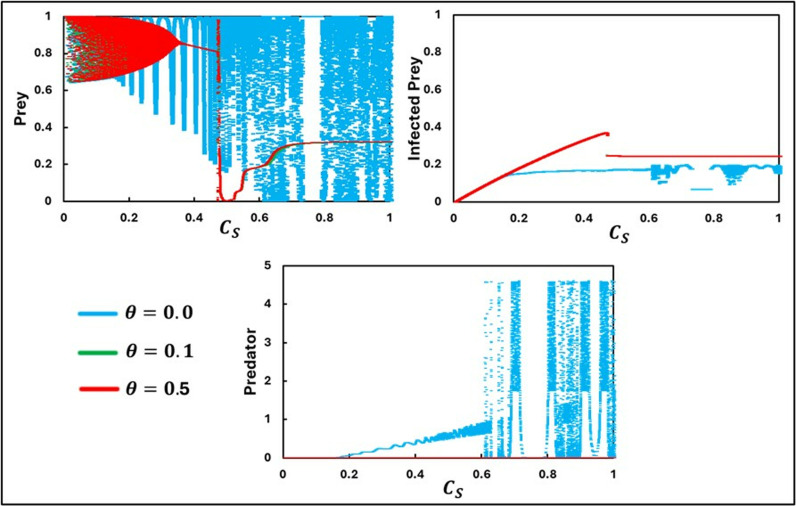
The figure presents a bifurcation diagram depicting equilibria, oscillatory dynamics, and chaotic behavior about the parameter $C_{S}$. The subpanels **(a)**, **(b)**, and (c) show the trends for Prey, Infected Prey, and Predator, respectively. Three different values for the Allee parameter θ are considered: θ=0.0, θ=0.1, and θ=0.5. The other parameters are set as follows: $a=0.3636,\;b=1.0,\;\alpha_{1}=0.01,\;\sigma =15,\;c=0.01,\;r=1.0,\;\alpha_{2}=0.05,\;d=0.5,\;\mu =0.4,\;c_{1}=2,\;\theta =0,\;c_{2}=1.0,\;e=1.0,\;C_{P}=0.5,\;$and  m=0.01 [47].

In Fig 8, we examine the behavior of the system along $C_{S}$ for three different values of the Allee parameter effect θ(0.0, 0.1, and 0.5). The results show a pattern like that observed in Fig 7, particularly concerning the impact of the Allee effect parameter θ and the cost $C_{S}$. One noteworthy observation pertains to the susceptible prey species. When the Allee effect is present, specifically for θ=0.1 and θ=0.5, we see an interesting dynamic as $C_{S}$ changes. At lower values of $C_{S}$, the susceptible prey population exhibits periodic oscillations. The susceptible prey population faces extinction as $C_{S}$ increases and crosses a certain threshold. However, beyond this threshold, further increases in $C_{S}$ lead to the re-emergence of the susceptible prey population. From an ecological perspective, these findings can be interpreted in several ways.

When $C_{S}$ is low, resources or environmental conditions are sufficient to support predator and susceptible prey populations, allowing them to coexist in a dynamic equilibrium. The periodic oscillations may represent natural cycles of predator-prey interactions, where predator populations rise and fall in response to susceptible prey availability and vice versa. As $C_{S}$ increases, indicating perhaps a rise in predation cost or environmental stress, the susceptible prey population cannot sustain itself, leading to extinction. This threshold effect highlights the vulnerability of prey populations to changes in environmental conditions or predation pressures, especially when an Allee effect is present. The Allee effect suggests that the susceptible prey population needs to be above a certain critical size to avoid extinction, and higher $C_{S}$ may prevent them from reaching this size. Surprisingly, further increases in $C_{S}$ allow the susceptible prey population to re-establish. This counterintuitive result might be due to complex ecological interactions or adaptive behaviors that emerge under extreme conditions. For instance, higher predation costs might reduce predator efficiency or numbers, relieving pressure on the susceptible prey population and allowing it to recover. In a real-world scenario, these dynamics could correspond to various ecological phenomena. Low $C_{S}$ could represent well-connected habitats where susceptible prey can find refuge and maintain their numbers. As habitats become fragmented (higher $C_{S}$), susceptible prey populations might initially decline due to increased isolation and predation. However, if predator movement is also restricted by fragmentation, susceptible prey might eventually find new equilibrium points where they can survive. Increasing $C_{S}$ could symbolize escalating environmental stress due to climate change or human activities. Initially, susceptible prey populations might struggle and face extinction. But over time, adaptations or changes in predator behavior could create conditions for prey recovery. Overall, these findings emphasize the importance of considering both the Allee effect and environmental costs in managing and conserving ecosystems. Understanding these dynamics can help predict species’ responses to changing conditions and guide effective conservation strategies.

**Fig 9:**
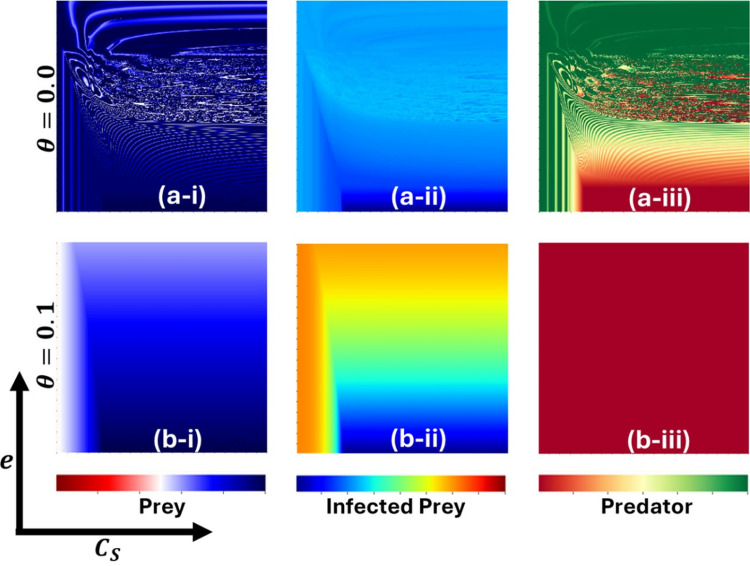
The figure displays a two-dimensional phase diagram illustrating the dynamics of (*-i) Prey, (*-ii) Infected Prey, and (*-iii) Predator as functions of $C_{S}$ and e for $\beta_{0}=0.7$. Subpanels (a-*) and (b-*) present the outcomes for the Allee parameters θ=0.0, and θ=0.1, respectively. The other parameters are set to $a=0.3636,\;b=1.0,\;\alpha_{1}=0.01,\;\sigma =15,\;c=0.01,\;r=1.0,\;\alpha_{2}=0.05,\;d=0.5,\;\mu =0.4,\;c_{1}=2,\;\theta =0,\;c_{2}=1.0,\;e=1.0,\;C_{P}=0.5,\;$and  m=0.01 [47].

**Fig 10:**
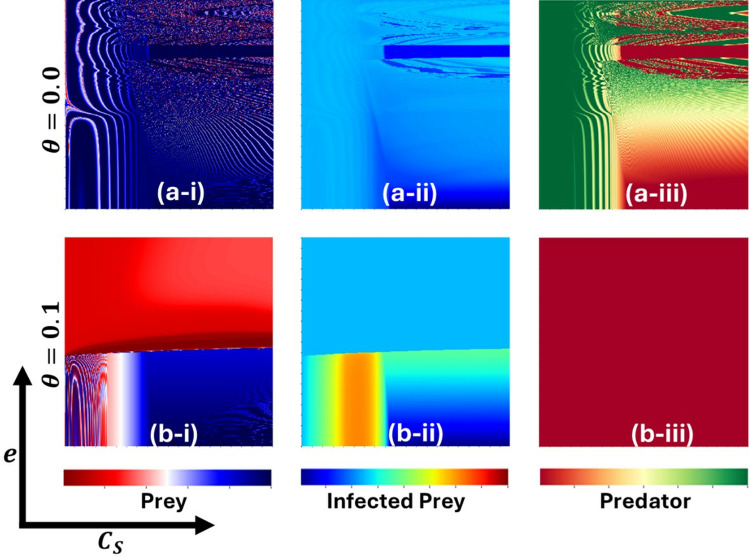
The figure displays a two-dimensional phase diagram illustrating the dynamics of (*-i) Prey, (*-ii) Infected Prey, and (*-iii) Predator as functions of $C_{S}$ and e for $\beta_{0}=0.95$. Subpanels (a-*) and (b-*) present the outcomes for the Allee parameters θ=0.0, and θ=0.1, respectively. The other parameters are set to $a=0.3636,\;b=1.0,\;\alpha_{1}=0.01,\;\sigma =15,\;c=0.01,\;r=1.0,\;\alpha_{2}=0.05,\;d=0.5,\;\mu =0.4,\;c_{1}=2,\;\theta =0,\;c_{2}=1.0,\;e=1.0,\;C_{P}=0.5,\;$and  m=0.01 [47].

Figs 9 and 10 elucidate critical parameters for managing epidemics under conditions where β equals 0.7 and 0.95, respectively. These figures focus on three categories: (*-i) Susceptible Prey, (*-ii) Infected Prey, and (*-iii) Predator. The insights presented are derived from variations in two pivotal parameters: the controlling cost associated with susceptible prey $\left(C_{S}\right)$ on the x−axis and the proportionality constant for cooperation (e) on the y−axis. Each figure examines two scenarios concerning the Allee effect parameter—(a-*) θ=0.0 and (b-*) θ=0.1. In Fig 9, for θ=0.0 (panel (a-)), susceptible prey exhibits oscillatory behavior (panel (a-i)). Predators demonstrate a survival region at higher values of e and lower values of $C_{S}$ (panel (a-iii)). However, when θ=0.1 (panel (b*-*)), susceptible prey presents a stable and sustainable population (panel (b-i)), while Predators face complete dissipation, indicating that no Predators survive (panel (b-iii)).

In Fig 10, Predators exhibit a survival region only at lower values of $C_{S}$ (panel (a-iii)), susceptible prey maintains stability when e is higher, though dissipation is also observed (panel (b-i)). At lower values of e, an oscillatory situation is observed for low values of $C_{S}$. In this scenario, Predators also fully dissipate, resulting in no Predator survival (panel (b-iii)). Infected prey survives in both cases when the Allee effect is higher. In contrast, infected prey is completely eradicated for lower Allee effect values. The influence of the parameters e and $C_{S}$ on Infected prey is also observed.

From an ecological perspective, these findings suggest that the Allee effect plays a significant role in population dynamics. The stability of susceptible prey populations under higher Allee effects (θ=0.1) implies a threshold density below which populations cannot sustain themselves. This has real-world implications for species conservation, particularly for those that rely on group behaviors for survival and reproduction.

The reduction of predator populations in environments with more substantial Allee effects reveals their vulnerability to cooperation and cost management mechanisms shifts. Such populations need a specific density to hunt and reproduce effectively, and the presence of infected prey surviving under these conditions suggests that diseases can persist when predators are less prevalent, thanks to lower pressures on the susceptible prey, allowing pathogens to endure despite fewer hosts. These insights underline the importance of considering cooperative behaviors and cost strategies in wildlife management and epidemic prevention, stressing the need to maintain critical population levels and understand the complex role of cooperation and cost controls in ecosystem balance.

## Conclusion

This study introduces a novel eco-epidemiological model that integrates behavioral dynamics into the predator-prey framework, explicitly incorporating infected prey populations. The model uniquely combines epidemic dynamics with prey-predator interactions, employing evolutionary game theory (EGT) to capture behavioral strategies such as cooperation and non-cooperation. A key innovation is the inclusion of time-varying parameters tied to infection duration distributions, enhancing the model’s applicability to both recurring diseases with existing immunization measures and emerging epidemics. Numerical simulations reveal that disease transmission, behavioral strategies, and ecological factors like the Allee effect interact to shape system dynamics, including equilibrium states, periodic oscillations, and transitions to instability. One of the most impactful findings is the pivotal role of non-linear behaviors and behavioral strategies in influencing infection rates and population stability. For instance, low disease transmission rates stabilize the system around a coexistence equilibrium, while higher rates lead to periodic oscillations and instability. The model highlights the critical influence of the Allee effect on prey populations, with higher impact of Allee effect exacerbating oscillatory behavior and increasing the vulnerability of susceptible prey. This dynamic interplay can ultimately eradicate infected prey while susceptible prey and predator populations continue oscillating. The proposed model offers significant insights into future studies by providing a framework to explore the complex interactions between ecological and epidemiological factors. Its ability to simulate real-world scenarios, such as the impact of behavioral changes or immunization strategies, positions it as a valuable tool for optimizing disease control measures, enhancing conservation efforts, and understanding the stability of ecosystems under infectious disease pressures. These contributions lay a foundation for developing more nuanced strategies in both ecological management and public health.

Additionally, the transitions occur at intermediate controlling costs of susceptible prey values, where susceptible prey’s increased investments in disease control disrupt the balance between reproduction, survival, and predation pressures. Biologically, this reflects trade-offs destabilizing population dynamics, leading to oscillations as the system responds to heightened disease prevalence and predation. Mathematically, these shifts correspond to bifurcations, such as Hopf bifurcations for periodic oscillations, and routes to chaos as cost increases further, driven by nonlinear feedback and changes in the stability of equilibrium points. These dynamics underscore the critical role of controlling costs for susceptible prey as a control parameter in shaping system behavior.

This analysis highlights how the Allee effect and transmission rate jointly influence the system’s stability, offering essential implications for understanding disease dynamics in ecological systems. A higher Allee threshold signifies greater susceptibility to extinction, as a larger critical population is required for growth. This has significant real-world implications for species conservation, particularly for those facing overhunting or habitat loss. Conversely, a lower Allee threshold indicates resilience, allowing populations to sustain growth at lower densities. The findings underscore the substantial impact of the Allee effect on the stability and persistence of susceptible prey, infected prey, and predator populations. The heightened Allee effect can precipitate predator extinction due to declining susceptible prey populations, which affects predators’ food sources. Conversely, reducing the disease transmission rate enhances susceptible prey population health, supporting more stable predator populations and a balanced ecosystem.

The study also reveals that reducing the disease transmission rate significantly enhances the health and stability of susceptible prey populations, supporting predator populations and fostering a balanced ecosystem. This insight provides decision-makers with a robust framework for developing targeted interventions, such as disease management programs, habitat restoration, or controlled population reductions for predators in overburdened systems. By integrating ecological and epidemiological factors into conservation strategies, this model enables the identification of thresholds and tipping points, ensuring proactive measures can be taken to prevent ecosystem collapse. Furthermore, this framework can inform policies for managing wildlife diseases, guiding vaccination campaigns, and optimizing resource allocation to protect vulnerable species. Its application to real-world scenarios, such as predicting the impacts of emerging diseases or human-induced ecological changes, enhances its utility for conservation biologists, ecologists, and public health officials. By bridging theoretical models with actionable strategies, this study provides a pathway for sustaining biodiversity and maintaining ecological balance in the face of ongoing environmental challenges.

## Supporting information

S1 File Supplementary mathematical derivations and additional results supporting the main findings are provided in the Appendix. (PDF)
