# Supplementary material for: Integrating behavioral dynamics and allee effect in eco-epidemiological model: A comprehensive approach to disease impact
Source: PLoS One. 2025 May 22;20(5):e0323928. doi: 10.1371/journal.pone.0323928 (PMC12097609; doi:10.1371/journal.pone.0323928)
Supplement: S1 File — (PDF) [file pone.0323928.s001.pdf]

Appendix

Boundedness

Lemma: All the solutions of the system (equations 1.1-1.3) that initiate in R^3 are uniformly bound.

Proof: Let, $U = S + I + P$.

$$\frac{dU}{dt} \leq (r - bS - cI)S - \mu I - mP$$

$$\Rightarrow \frac{dU}{dt} + \eta U \leq (r + \eta - bS)S + (\eta - \mu)I + (\eta - m)P$$

Here, $\eta = \min\{\mu, m\}$

Therefore, $\frac{dU}{dt} + \eta U \leq -b\left[S^2 - 2\left(\frac{r+\eta}{2b}\right)S + \left(\frac{r+\eta}{2b}\right)^2\right] + \frac{(r+\eta)^2}{4b}$,

$$\Rightarrow \frac{dU}{dt} + \eta U \leq -b\left(S - \left(\frac{r+\eta}{2b}\right)\right)^2 + \frac{(r+\eta)^2}{4b}$$

According to the theory of differential inequality,

$$0 < U(S, I, P) \leq \frac{(r+\eta)^2}{4b\eta} + e^{-\eta t}\left[U(0,0,0) - \frac{(r+\eta)^2}{4b\eta}\right],$$

For, $t \to \infty, 0 < U \leq \frac{(r+\eta)^2}{4b\eta}$, implies, $\frac{dU}{dt} + \eta U \leq \frac{(r+\eta)^2}{4b}$.

Hence the system is bounded.

Positiveness

From equations (1.1-1.3), we can obtain,

$$\frac{dS}{dt} \leq [r - bS]S,$$

$$\frac{dI}{dt} \leq \beta SI - \frac{\alpha_2 IP}{d + I} - \mu I,$$

$$\frac{dP}{dt} \leq c_1\alpha_1 SP + c_2\alpha_2 IP - mP. \qquad \text{(A1)}$$

We get, $\lim_{t\to\infty} \sup S(t) \leq \frac{r}{b} = u_1$.

From second equation of (A1),

$$\frac{dI}{dt} \leq \left[\beta S - \frac{\alpha_2 P}{d + I} - \mu\right] I$$

$$\le \left[\frac{\left(\frac{\beta rd}{b} - \mu d - \alpha_2 P\right) - \left(\mu - \frac{\beta r}{b}\right) I}{d + I}\right] I$$

Finally,

$$\lim_{t\to\infty} \sup I(t) \le \frac{(\beta rd - \mu bd - \alpha_2 bP)}{(b\mu - \beta r)}.$$

And $\frac{dP}{dt} \le P[c_1\alpha_1 S + c_2\alpha_2 I - m]$

$$\le P\left[\frac{c_1\alpha_1 r}{b} - m - c_2 d\alpha_2 - \frac{c_2\alpha_2^2 bP}{b\mu - \beta r}\right]$$

Again, $\lim_{t\to\infty} \sup P(t) \le \frac{(\beta r - b\mu)(bm + bdc_2\alpha_2 - c_1\alpha_1 r)}{b^2 c_2 \alpha_2^2}$

Using it, we get, $\lim_{t\to\infty} \sup I(t) \le \frac{bm - c_1\alpha_1 r}{bc_2\alpha_2}$

Lemma. If $bm > c_1\alpha_1 r$, $\beta r > b\mu$, then the system is positive.

Existence of the equilibrium points

Analysis of system's equilibrium points

In this section, we compute and analyze the equilibrium points of the system. We examine the conditions under which these equilibrium points exist and determine the types of stability associated with each point. Below, we present and detail five potential equilibrium points. The Jacobian matrix J is,

$$J = \begin{bmatrix} r - 2bS - cI - \frac{a\beta I}{(a+S)^2} - \frac{e\alpha_1 P}{(\sigma+S)^2} & \frac{a\beta I}{(a+S)^2} & \frac{e\alpha_1 c_1 P^2}{(a+S)^2(\theta+P)} \\ -cS - \frac{\beta S}{a+S} & \frac{\beta S}{a+S} - \frac{d\alpha_2 P}{(d+I)^2} - \mu & \frac{d\alpha_2 c_2 P^2}{(d+I)^2(\theta+P)} \\ -\frac{\alpha_1 S}{\sigma+S} & -\frac{\alpha_2 I}{d+I} & \frac{(2\theta+P)P}{(\theta+P)^2}\left(\frac{c_1\alpha_1 S}{\sigma+S} + \frac{c_2\alpha_2 I}{d+I}\right) - m \end{bmatrix}$$

Point 1: The trivial equilibrium point $M_1 = (0,0,0)$ signifies the absence of populations in the model. The eigenvalues about M_1 are $r, -\mu, -m$. Hence M_1 is a saddle point and has unstable manifold along x −axis.

Point 2: The axial equilibrium point $M_2 = (\frac{r}{b}, 0,0)$ indicates the presence of only susceptible prey in the model. The eigenvalues of M_2 are $-r, \frac{\beta r}{ab+r} - \mu, -m$. Thus, M_2 will be locally asymptotically stable when $\frac{\beta r}{ab+r} - \mu < 0$ i.e. $r < \frac{\mu ab}{\beta - \mu}$. It has been determined that M_2 is saddle when $r > \frac{\mu ab}{\beta - \mu}$.

Point 3: The planar equilibrium point $M_3 = (\frac{a\mu}{\beta-\mu}, \frac{a(r\beta - r\mu - ab\mu)}{(\beta-\mu)(\beta+ac-\mu)}, 0)$ signifies the coexistence of susceptible and infected prey populations in the model.

One of the eigenvalues corresponding to the equilibrium point M_3 is $-m$. The other two eigenvalues of the equilibrium point M_3 is given by $\lambda^2 - \psi_1\lambda + \psi_2 = 0$. For the values of,

$x_2 = \frac{a\mu}{\beta-\mu}$ and $y_2 = \frac{a(r\beta - r\mu - ab\mu)}{(\beta-\mu)(\beta+ac-\mu)}$,

$$\psi_1 = r - 2bx_2 - cy_2 - \frac{a\beta y_2}{(a+x_2)^2} + \frac{\beta x_2}{a+x_2} - \mu,$$

$$\psi_2 = \left(r - 2bx_2 - cy_2 - \frac{a\beta y_2}{(a+x_2)^2}\right)\left(\frac{\beta x_2}{a+x_2} - \mu\right) + \frac{a\beta y_2}{(a+x_2)^2}\left(cy_2 + \frac{\beta x_2}{a+x_2}\right),$$

Thus, it can be confirmed that M_3 is locally asymptotically stable when, $\psi_1 < 0, \psi_2 < 0$ i.e. $r < \frac{\mu \text{ab}}{\beta-\mu} \frac{3\beta^2+\mu^2+ac\beta-4\beta\mu}{2\beta^2+\mu^2-3\beta\mu}$.

Point 4: The planar equilibrium point $P_4 = (S^*, 0, \frac{bc_1\sigma S^* - \sigma c_1 r - m\theta}{m - c_1\alpha_1})$ indicates the model's coexistence of susceptible prey and predator populations. Here, S^* represents the following equation's non-negative root(s).

$$(mb - bc_1\alpha_1){S^*}^2 + (\sigma mb + rc_1\alpha_1 - mr)S^* - \sigma mr - m\theta\alpha_1 = 0.$$

Let, $A = mb - bc_1\alpha_1$, $B = \sigma mb + rc_1\alpha_1 - mr$, and $C = -\sigma mr - m\theta\alpha_1$, that represent,

$$A{S^*}^2 + BS^* + \text{C} = 0.$$

The system has, (i) no disease free equilibrium for $B^2 < 4AC$, (ii) single disease free equilibrium for $B^2 = 4AC, A < 0$ and $\text{B} > 0$ or $B^2 = 4AC, A > 0$ and $\text{B} < 0$, (iii) double disease free equilibria for $B^2 > 4AC, A < 0,$ $\text{B} > 0$ and $\text{C} < 0$, or $B^2 > 4AC, A > 0,$ $\text{B} < 0$ and $\text{C} > 0$, and (iv) one positive disease free equilibrium for $B^2 > 4AC, A < 0,$ and $\text{C} > 0$ or $B^2 > 4AC, A > 0,$ and $\text{C} < 0$.

Additionally, the quadratic equation must have a negative solution. The corresponding characteristic equation around this equilibrium point is computed as follows.

$\lambda^3 + L_1\lambda^2 + L_2\lambda + L_3 = 0,$ where, $L_1 = -(w_1 + w_4 + w_7), L_2 = -(w_1w_7 + w_4w_7 + w_1w_4)$, $L_3 = (w_1w_7 - w_2w_6)w_4$, and $w_1 = r - 2bS^* - \frac{\sigma\alpha_1 S^*}{(\sigma+S^*)^2}$, $w_2 = \frac{\sigma\alpha_1 c_1 {\text{P}^*}^2}{(\theta+\text{P}^*)(\sigma+S^*)^2}$, $w_3 = -cS - \frac{\beta S^*}{a+S^*}, w_4 = \frac{\beta S^*}{a+S^*} - \frac{\alpha_2 \text{P}^*}{\text{d}} - \mu$, $w_5 = \frac{\alpha_2 c_2 {\text{P}^*}^2}{d(\theta+\text{P}^*)}$, $w_6 = -\frac{\alpha_1 S^*}{\sigma+S^{**}}$, $w_7 = -\frac{\alpha_1 c_1 S^* \text{P}^*(2\theta+\text{P}^*)}{(\sigma+S^*)(\theta+\text{P}^*)^2} - \text{m}$.

The equilibrium will be locally asymptotically stable for $w_1 + w_4 + w_7 < 0$; $w_1w_7 + w_4w_7 + w_1w_4 < 0$; $(w_1w_7 - w_2w_6)w_4 > 0$; and $(w_1 + w_4 + w_7)(w_1w_7 + w_4w_7 + w_1w_4) > (w_1w_7 - w_2w_6)w_4$.

Point 5: The interior equilibrium point is $P_5 = (S^*, \mathrm{I}^*, \mathrm{P}^*)$. These components must adhere to the following relationship.

$$r - bS^* - \mathrm{c}\mathrm{I}^* - \frac{\beta \mathrm{I}^*}{a + S^*} - \frac{\alpha_1 \mathrm{P}^*}{\sigma + S^*} = 0,$$

$$\frac{\beta S^*}{a + S^*} - \frac{\alpha_2 P^*}{d + I^*} - \mu = 0,$$

$$\left(\frac{c_1 \alpha_1 S^*}{\sigma + S^*} + \frac{c_2 \alpha_2 I^*}{d + I^*}\right)\frac{P^*}{\theta + P^*} - m = 0$$

By solving this system of equations, we obtain two positive interior equilibrium points: $P_5^1 =$ $= (S_1^*, I_1^*, P_1^*)$ and $P_5^2 = (S_2^*, I_2^*, P_2^*)$. However, due to the complexity of the mathematical expressions, we have not explicitly derived the formulas for these equilibrium points.

The analysis shows that the equilibrium point P_5 is locally asymptotically stable when the roots of the characteristic cubic equation, derived from the Jacobian matrix, meet the necessary stability conditions. Specifically, for the interior equilibrium point P_5, the characteristic equation governing the local dynamics around P_5 is given by, $\lambda^3 + B_1\lambda^2 + B_2\lambda + B_3 = 0$. According to the Routh-Hurwitz stability criteria, the equilibrium point P_5 is locally asymptotically stable if the following conditions are satisfied, $B_i > 0,\ B_1 B_2 > B_3$. These criteria ensure that all the roots of the characteristic equation have negative real parts, which is a key requirement for local asymptotic stability. Thus, when these conditions hold, the system dynamics near the equilibrium point P_5 will tend to return to equilibrium after small perturbations, confirming that P_5 is locally asymptotically stable. The Jacobian matrix J is,

$$J = \begin{bmatrix} r - 2bS^* - cI^* - \frac{a\beta I^*}{(a + S^*)^2} - \frac{e\alpha_1 P^*}{(\sigma + S^*)^2} & \frac{a\beta I^*}{(a + S^*)^2} & \frac{e\alpha_1 c_1 (P^*)^2}{(a + S^*)^2(\theta + P^*)} \\ -cS^* - \frac{\beta S^*}{a + S^*} & \frac{\beta S^*}{a + S^*} - \frac{d\alpha_2 P^*}{(d + I^*)^2} - \mu & \frac{d\alpha_2 c_2 (P^*)^2}{(d + I^*)^2(\theta + P^*)} \\ -\frac{\alpha_1 S^*}{\sigma + S^*} & -\frac{\alpha_2 I^*}{d + I^*} & \frac{(2\theta + P^*)P^*}{(\theta + P^*)^2}\left(\frac{c_1\alpha_1 S^*}{\sigma + S^*} + \frac{c_2\alpha_2 I^*}{d + I^*}\right) - m \end{bmatrix}$$

Now, let,

$$J = \begin{bmatrix} b_{11} & b_{12} & b_{13} \\ b_{21} & b_{22} & b_{23} \\ b_{31} & b_{32} & b_{33} \end{bmatrix}$$

and

$B_1 = -b_{11} - b_{22} - b_{33},$

$B_2 = b_{11}b_{22} + b_{22}b_{33} + b_{11}b_{33} - b_{12}b_{21} - b_{13}b_{31} - b_{23}b_{32},$

$B_3 = b_{11}b_{23}b_{32} + b_{12}b_{21}b_{33} + b_{13}b_{31} - b_{11}b_{22}b_{33} - b_{12}b_{31}b_{23} - b_{13}b_{21}b_{32},$

Theorem (Global Stability): The positive interior equilibrium $P_5 = (S^*, \mathrm{I}^*, \mathrm{P}^*)$ is globally asymptotically stable when $c_1 > c_1$,

$$c_1 b\left(\frac{r+bS^*}{2b}\right)^2 - c_1 rS^* + c_1\frac{US^*}{a+K} + c_1\frac{\alpha_1 US^*}{\sigma+K} + \frac{\alpha_2 UI^*}{d+K} + c_2\mu I^* + mP^*$$

$$< c_1 rS^* + c_2\frac{\beta KI^*}{a+U} + P^*\left(c_1\frac{\alpha_1 K}{\sigma+U} + c_2\frac{\alpha_2 K}{d+U}\right)\frac{K}{\theta+U}.$$

Proof: Let, $L(S,I,P) = L_1(S,I,P) + L_2(S,I,P) + L_3(S,I,P)$.

Here, $L_1(S,I,P) = c_1\left[S - S^*\ln\left(\frac{S}{S^*}\right)\right]$,

$$L_2(S,I,P) = c_2\left[I - I^*\ln\left(\frac{I}{I^*}\right)\right],$$

$$L_3(S,I,P) = P - P^*\ln\left(\frac{P}{P^*}\right).$$

The function L is shown to be a Lyapunov function. It is noted that $L(S,I,P)$ vanishes at P_5 and remains positive for all $S,I,P > 0$. As a result, P_5 is identified as the global minimum.

$$\frac{dL}{dt} = \frac{c_1(S-S^*)}{S}\frac{dS}{dt} + \frac{c_2(I-I^*)}{I}\frac{dI}{dt} + \frac{(P-P^*)}{P}\frac{dP}{dt}.$$

Since the system is persistent, it can be assumed, without loss of generality, that two constants, U and K, exist such that $S(t), I(t), P(t) < U$ and $S(t), I(t), P(t) > K$, respectively. After performing some algebraic calculations, the following expression is obtained:

$$\frac{dL}{dt} \le c_1(S-S^*)(r - bS - cI) + (c_2 - c_1)\frac{\beta SI}{a+S} + c_1\frac{IS^*}{a+S} + c_1\frac{\alpha_1 PS^*}{\sigma+S} - c_2\frac{\beta SI^*}{a+S}$$
$$+ \frac{\alpha_2 PI^*}{d+I} - c_2\mu(I-I^*) - m(P-P^*) - P^*\left(c_1\frac{\alpha_1 S}{\sigma+S} + c_2\frac{\alpha_2 I}{d+I}\right)\frac{P}{\theta+P}.$$

Thus,

$$\frac{dL}{dt} \le -c_1 b\left(S - \frac{r+bS^*}{2b}\right)^2 + c_1 b\left(\frac{r+bS^*}{2b}\right)^2 - c_1 rS^* + c_1\frac{US^*}{a+K} + c_1\frac{\alpha_1 US^*}{\sigma+K} + \frac{\alpha_2 UI^*}{d+K}$$
$$- c_2\frac{\beta KI^*}{a+U} - (c_1 - c_2)\frac{\beta SI}{a+S} - c_2\mu(I-I^*) - m(P-P^*)$$
$$- P^*\left(c_1\frac{\alpha_1 K}{\sigma+U} + c_2\frac{\alpha_2 K}{d+U}\right)\frac{K}{\theta+U}.$$

It can be readily confirmed that $\frac{dL}{dt} < 0$. Therefore, the function L serves as a Lyapunov function within the interior of the positive octant concerning P_5. As a result, the equilibrium point $P_5 = = (S^*, \mathrm{I}^*, \mathrm{P}^*)$ is globally asymptotically stable in $\mathbb{R}^3_+$.
